# Immunoglobulin A vasculitis with aneurysmal subarachnoid hemorrhage in an adult

**DOI:** 10.1016/j.jdcr.2025.07.034

**Published:** 2025-09-06

**Authors:** Priya Patel Housley, Caitlin G. Purvis, Brittany Liebenow, Thiago Santos Carneiro, Nohra Chalouhi, Christopher Robinson, Kiran Motaparthi

**Affiliations:** a College of Medicine, University of Florida, Gainesville, Florida; b Department of Dermatology, College of Medicine, University of Florida, Gainesville, Florida; c Department of Neurosurgery, University of Florida, Gainesville, Florida; d Department of Neurology, University of Florida, Gainesville, Florida

**Keywords:** cerebral vasculitis, Henoch-Schönlein purpura, IgA vasculitis, intracranial hemorrhage, subarachnoid hemorrhage

## Introduction

Immunoglobulin A vasculitis (IgAV), also known as Henoch-Schönlein purpura, is a small-vessel vasculitis characterized by IgA immune complex deposition in vascular intima. Clinical manifestations include palpable purpura, arthralgia, abdominal pain, and renal involvement. While children are predominantly affected, cases involving adults are associated with increased symptom severity.^1^

Central nervous system (CNS) is rare, occurring in 2% to 8% of patients. Seizures are the most frequently reported symptom, affecting over half of these patients.^2^ Infrequently, more severe CNS involvement includes cerebral vasculitis, hemorrhage, and posterior reversible encephalopathy syndrome.^2^ Of these complications, intracerebral and intracranial hemorrhage are particularly rare.^3^^,^^4^ We present a case of IgAV in a woman with CNS involvement manifesting as subarachnoid hemorrhage (SAH) secondary to a ruptured aneurysm.

## Case presentation

A 46-year-old woman presented to the emergency department with 1 week of headache and sudden onset of syncope. A review of systems noted arthralgia and gastrointestinal symptoms. At presentation, the patient was normotensive (130/79 mmHg). Physical exam revealed resolving palpable purpura. Although the neurologic exam was unremarkable at the time of evaluation, the patient reported word-finding difficulty earlier. Based on this history, computed tomography (CT) was performed, revealing an acute SAH in the interhemispheric fissure and bilateral sylvian fissures. The patient was admitted to an intensive care unit.

One month earlier at an outside hospital, she received a clinical diagnosis of a small vessel vasculitis after she presented with diarrhea, emesis, diffuse rash, and arthralgia. Her blood pressure at the outside hospital was unknown, a biopsy for direct immunofluorescence (DIF) was not performed, and no medications, including systemic steroids, were prescribed.

**Fig 1:**
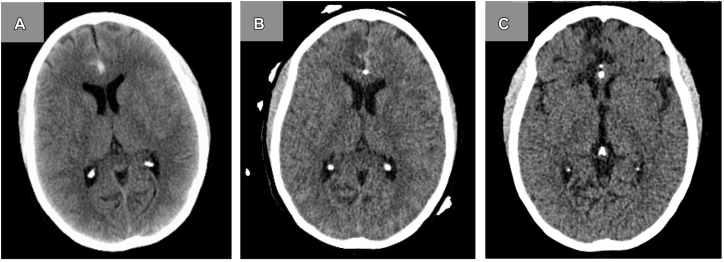
Serial noncontrast CT axial head images. **A,** Initial presentation with diffuse subarachnoid hemorrhage within an interhemispheric fissure. **B,** One day postoperative following stent and coil embolization of 2 right A2-3 junction aneurysms. Decreased density is consistent with the evolution of clot. **C,** Eight-week follow-up showing chronic infarction of ACA territory. *ACA*, Anterior cerebral artery; *CT*, computed tomography.

During her intensive care course following the SAH, arterial line blood pressures were elevated with readings up to 228/105 mmHg. Despite preserved renal function, there was evidence of renal involvement including hematuria and proteinuria. CT (Fig 1) revealed SAH secondary to 2 right A2-3 junction aneurysms, and she underwent stent and coil embolization of both. Intraoperatively, stent thrombosis was noted, and the patient was started on antiplatelets. Her postoperative course was complicated by anterior cerebral artery (ACA) territory infarcts and bilateral supplementary motor area syndrome. She underwent SAH protocol and had severe vasospasm. During treatment for vasospasm, angioplasty was attempted for the stented region, but thrombosis occurred, resulting in a right ACA infarct.

**Fig 2:**
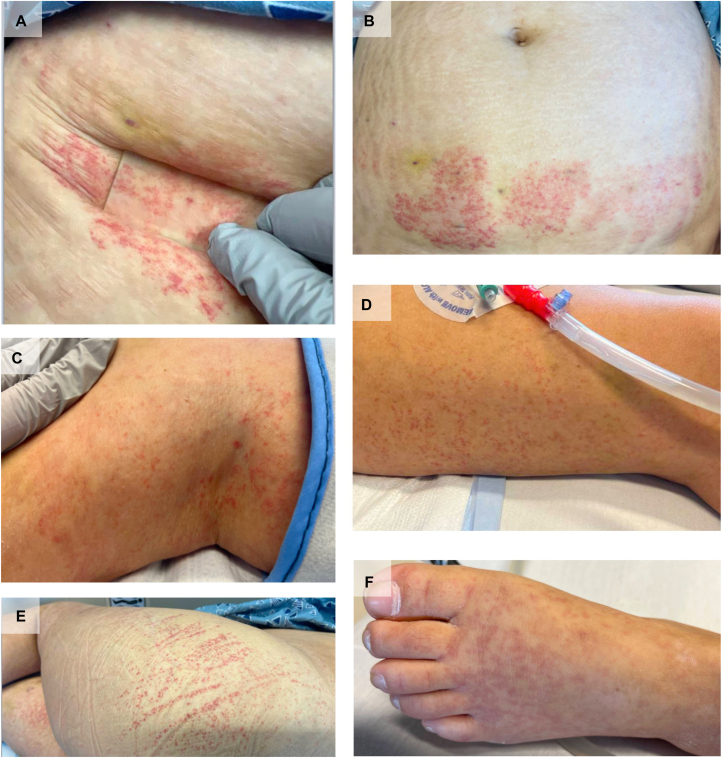
Skin findings during hospitalization. Purpuric papules and macules on the abdomen **(A** and **B),** lower extremities **(C**-**E),** and foot **(F)**.

**Fig 3:**
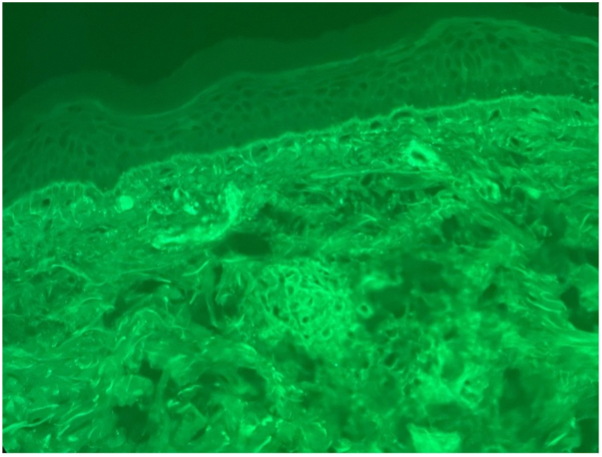
Strong granular deposition of IgA within small vessels of the superficial dermis (direct immunofluorescence with IgA, 400× magnification).

Because of the resolving leukocytoclastic vasculitis, serologic workup was performed for systemic vasculitides associated with both small-medium vessel disease and CNS involvement. Serologic studies including serum protein electrophoresis, complement levels (C3 and C4), rheumatoid factor, cryoglobulins, antinuclear antibody, antiphospholipid panel, and anti-neutrophil cytoplasmic antibody were all negative. While admitted for these neurologic sequelae, new palpable purpura reappeared on the lower extremities, trunk, and arms (Fig 2). Biopsy for DIF confirmed IgAV (Fig 3). Treatment was delayed due to a lack of evidence linking IgAV to the patient’s current neurological findings, given that DIF was not performed at onset 1 month earlier and because palpable purpura was absent at admission for neurologic findings. Improvement was noted following 5 days of plasma exchange and intravenous methylprednisolone 1 mg/kg/day. Rituximab 1 g intravenous was administered at day 0 and day 15. Upon discharge, the patient was transitioned to oral prednisone tapered over 6 weeks.

The patient remains under the care of outpatient dermatology and neurosurgery. She is now ambulatory without a cane, independently performs activities of daily living, and is preparing to return to work. She remains on rituximab maintenance treatment every 6 months. CT at 2 and 4 months after discharge demonstrated stability of her chronic right ACA infarction without hemorrhage or hydrocephalus.

## Discussion

CNS involvement with IgAV is uncommon, with intracranial hemorrhage being especially rare.^5^ This example of SAH due to IgAV in an adult reflects a rare complication in an atypical population.^2^

Common signs of IgAV with intracranial hemorrhage include focal neurological deficits, increased intracranial pressure, seizures, headache, changes in mental status, and behavioral changes.^6^ A 2003 review found only sporadic cases of SAHs or subdural hematomas, all in children.^3^ Most hemorrhages are intraparenchymal, often in the parieto-temporal region.^3^ While intracerebral hemorrhage is the most catastrophic CNS complication, the pathogenesis of cerebral vasculitis remains unclear. In reported cases associated with aneurysms, they were considered incidental.^3^ In our patient, her family history of aneurysms may suggest a predisposition to severe neurologic sequelae due to IgA CNS vasculitis, such as cerebral vasospasms and ACA infarct.

In all but 1 reported case, a latency of 1-4 days has been noted between the onset of typical IgAV symptoms and intracranial bleeding, but findings do not have to be concurrent with palpable purpura.^3^ This patient developed palpable purpura, arthralgias, and gastrointestinal symptoms 1 month before SAH. For early detection of CNS injury, electroencephalogram examination is crucial. For patients with IgAV and new-onset altered mental status, seizures, or focal deficits, prompt CT and magnetic resonance imaging are recommended to determine the presence of cerebral hemorrhage, posterior reversible encephalopathy syndrome, or other manifestations of neurologic involvement.

Renal involvement is noted in 30% to 50% of patients with IgAV and manifests with hematuria and proteinuria; however, renal manifestations can present asynchronously with skin involvement.^2^ Therefore, a DIF should be performed in all patients with IgAV, even if the tetrad of classic symptoms is present. If asynchronous but severe systemic findings present later or if uncommon sites of involvement (lungs and CNS) are suspected, a prior confirmatory DIF result can support treatment without delay.

IgA immune complex deposition in cerebral capillaries may initiate arteriolar inflammation, and cerebral hemorrhage may be secondary to refractory hypertension and cerebral vasculitis.^6^ Abnormal urinalysis and hypertension may precede neurologic findings.^6^

Emergent neurosurgery is recommended for cerebral intraparenchymal hemorrhage in IgAV.^2^ In this case of SAH, the patient underwent stent and coil embolization. Treatment for IgAV with renal involvement includes systemic corticosteroids.^5^ This patient also received plasma exchange and rituximab, which may induce disease remission and reduce steroid dependency.^7^ Most patients with IgAV and CNS involvement recover, as neurologic changes such as ischemia and encephalopathy are reversible. However, approximately 20% have permanent sequelae, and over half of those cases involve intracranial hemorrhage, which this patient experienced as part of her disease course.^8^

## Conflicts of interest

None disclosed.
